# Changes in bone density, microarchitecture, and biomechanical properties after plate removal in surgically treated distal radius fractures: a prospective study

**DOI:** 10.1007/s00264-025-06529-w

**Published:** 2025-04-14

**Authors:** Arastoo Nia, Stefan Hajdu, Gerhild Thalhammer, Silke Aldrian, Domenik Popp, Lukas Schmölz, Thomas Haider, Dieter Pahr, Alexander Synek

**Affiliations:** 1 University Clinic for Orthopedic and Trauma Surgery, Medical University of Vienna, General Hospital of Vienna, Vienna, Austria; 2 Department of Medicine, Danube Private University, Krems, Austria; 3 Institute of Lightweight Design and Structural Biomechanics, TU Wien, Vienna, Austria

**Keywords:** Radius fracture, HR-pQCT, Fracture healing, Bone mineral density, micro–Finite Element Analysis

## Abstract

**Purpose:**

Removal of volar locking plates after healing of a distal radius fracture is becoming increasingly common. However, it is unclear how the fracture healing proceeds and which defects remain. The aim of this study was to assess changes in bone microarchitecture and biomechanical properties in surgically treated radius fracture after volar locking plate removal.

**Methods:**

Twelve patients were recruited after undergoing plate removal. High Resolution Quantitative Computed Tomography (HR-pQCT) was used to perform scans of the fractured and contralateral distal radius on average one (M1) and 16 months (M2) after plate removal. Parameters measured were cortical- (Dcomp), trabecular- (Dtrab) and total bone density (D100), as well as cortical thickness (Ct.Th). Axial bone stiffness (FE.Kaxial) was determined through linear micro-finite element analysis (µFEA).

**Results:**

At M1, no significant differences between fractured and contralateral side were detected except for Dcomp. At the fractured side, all parameters except for Dtrab increased significantly between M1 and M2. At M2, Ct.Th and FE.Kaxial were significantly higher at the fractured side compared to the contralateral side, but Dcomp remained significantly lower. Qualitatively, closure of the screw holes was observed between M1 and M2, while large trabecular defects remained.

**Conclusion:**

Bone (re)modeling at the distal radius is an ongoing process even after plate removal and leads to a partial exaggeration of the bone properties relative to the intact contralateral side. It seems that the bone regains its biomechanical competence by closing screw holes and increasing cortical thickness, which compensates for trabecular defects that cannot be repaired.

**Level of evidence:**

III.

## Background

Distal radius fractures are among the most common types of fractures in adults [1]. In an analysis of the national Austrian Hospital Discharge Register (AHDR) and data from the Austrian Workers’ Compensation Board (AUVA), the incidence of distal forearm fractures in 2010 was 162/100,000 person-years for men and 607/100,000 person-years for women [2]. 

Surgical treatment should be considered for cases involving secondary dislocation, open fractures, unstable fractures—particularly comminuted or flexion fractures with dislocation—as well as intra-articular fractures with displacement and fractures that cannot be reduced. In 2010, Lichtman et al. provided a moderate recommendation for surgical management of distal radius fractures with a dorsal tilt exceeding 10°, radial shortening greater than 3 mm, and an articular step-off of more than 2 mm post-repositioning [3]. Volar plate osteosynthesis has now become established, whereas the use of the dorsal approach is less common [4]. 

Although assessment of bony changes after fracture treatment is a central task with therapeutic relevance in clinical practice, there is no gold standard for its implementation [5]. Routine diagnostics established clinically and in the context of studies include the clinical examination and the performance of radiographs [6]. The evaluation of the healing process of stable and unstable distal radius fractures using a combination of high-resolution peripheral quantitative computed tomography (HR-pQCT) and micro-finite element analysis (µFEA) has also already been reported in the literature. For instance, de Jong et al. examined 14 postmenopausal patients over a period of two years during conservative treatment of stable fractures [7].

The rate of plate removal is still unclear in the literature, it varies from 0 to 100% and the reasons for the removal are controversial, mostly due to missing symptoms of patients [8]. Nevertheless, studies show that stress shielding might weaken the bone and it remains unclear whether screw holes might pose a potential vulnerability for refractures. Studies about plate removal mostly cover the timepoint right after plate removal and, therefore leave this question open [9]. 

Recent studies put the refracture rate on the diaphyseal forearm after plate removal at 6.3 and 12.9% [10, 11]. However, this type of complication appears to be rare in the distal radius [12]. Yamamoto et al. analyzed 52 studies on the removal of volar locking plates from 19 countries as part of a review and reported a refracture rate of 1%, although it is unclear whether these complications occurred during removal or only during the course of the procedure [13]. This could be an indication that the implant-associated loss of bone density is more pronounced in the area of the diaphysis than at the distal radius and contributes to instability in addition to the residual screw holes, especially as the observed refractures often occur in the form of low-radius trauma in young patients [14]. However, there is currently no study that provides mid to long term data on the changes of the microarchitecture, bone density and biomechanics of unstable distal radius fractures after volar plate osteosynthesis and their removal.

The aims of this study were to investigate (I) midterm changes of radial bony microarchitecture and biomechanical properties after volar plate removal, and (II) differences of assessed parameters compared to the healthy contralateral radius.

## Methodology

### Study protocol

**Fig. 1:**
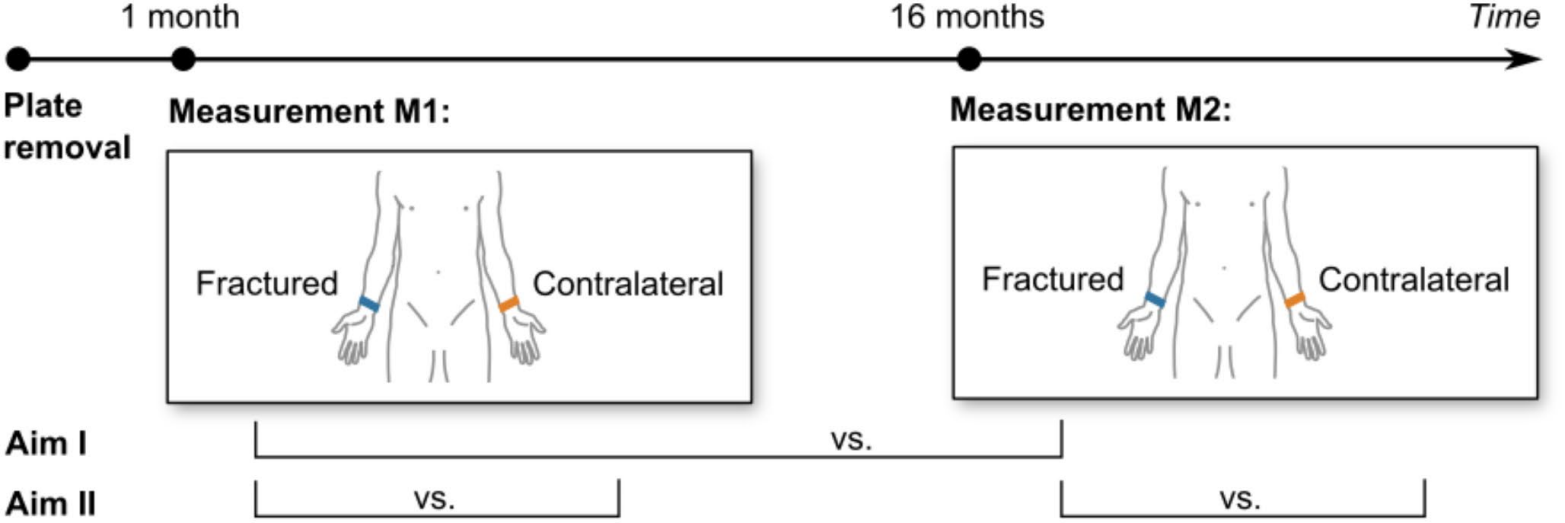
Study protocol

The study protocol and measurement workflow is shown in Fig. 1. and 2. Patients with a surgically treated unstable distal radius fracture were recruited at the level one orthopaedic surgery and traumatology.centre All fractures were surgically treated by volar plate osteosynthesis and all hardware was removed in a second surgery. Exclusion criteria were known systemic or metabolic disorders leading to progressive bone deterioration; use of glucocorticoids; presence of an active inflammatory disease; presence of an active or suspected infection; and malignancy in the last 12 months pre-fracture. Nicotine consumption was also recorded, since the effect of cigarette smoking on bone metabolism has been reported in many studies, although showing no statistically significant influence [15]. 

Patients were followed over an average of 15 month at two outpatient visits: Visit 1 (M1) and visit 2 (M2) post plate removal surgery (Fig. 1). HR-pQCT scans were taken at the same region of the distal radius at M1 and M2, both of the fractured and contralateral radius. The protocol was approved by the medical ethical review committee and all patients provided written informed consent before participation.

**Fig. 2:**
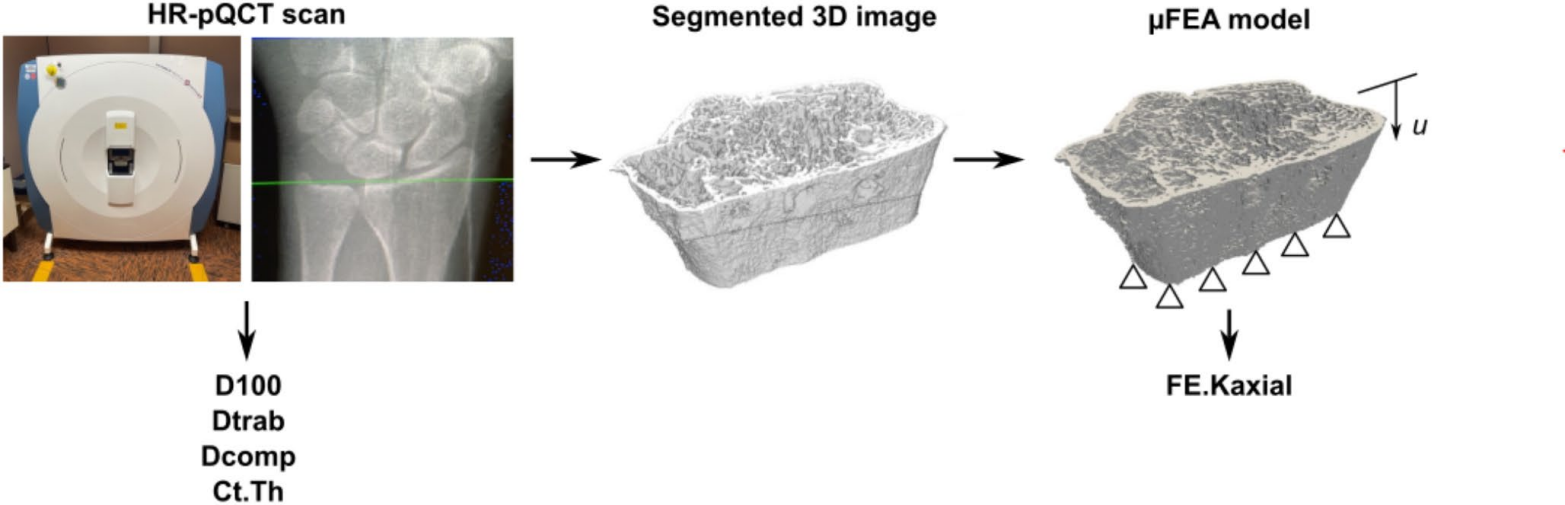
Processing workflow of the HR-pQCT scans. For a definition of the target parameters D100, Dtrab, Dcomp, Ct.Th and FE.Kaxial see Table 1

#### HR-pQCT scanning

At M1 and M2 the fractured radius as well as the healthy contralateral radius were scanned using an HR-pQCT scanner (XtremeCT, Scanco Medical AG, Brüttisellen, Switzerland) (Fig. 2). Settings were selected following the recommendations of the manufacturer for in vivo scanning. Tube voltage was 60 kVp and the tube current 900 µA. The region of interest was based on anteroposterior and lateral radiographs of the fractured radius, in which the proximal edge of the lunate was used as reference. The scan height was set to 9.02 mm. With an isotropic voxel size of 82 μm, each HR-pQCT measurement therefore resulted in 110 parallel CT slices. The forearm was placed in a cylindrical carbon holder with an inflatable cushion (Pearltec AG, Schlieren, Switzerland) to minimize patient motion. Scan quality was graded according to Pialat et al. [16], a five-grade scheme ranging from grade 1 (no motion artifacts) to grade 5 (severe motion artifacts). Scans with insufficient quality (grade 4 or 5) were repeated.

#### Density and microstructural parameters

Due to the limited resolution of the first-generation XtremeCT scanners and unknown influence of plate osteosynthesis and post removal screw holes on the measured values, target parameters were deliberately selected, which were considered to be robust (Table 1): Average bone density in the entire radius section (D100), trabecular (Dtrab) and cortical bone compartments (Dcomp), and the average cortical thickness (Ct.Th). These parameters showed good to very good correlations in comparative studies with the first and second generation of XtremeCT scanners [17, 18]. All parameters were evaluated using Scanco inhouse Software, which provides a semiautomatic contouring algorithm [19]. 

**Table 1:** Target parameters with definitions

Target parameters	Definition (unit)
D100	Total bone density (mgHA/cm³)
Dtrab	Trabecular bone density (mgHA/cm³)
Dcomp	Cortical bone density (mgHA/cm³)
Ct.Th	Cortical thickness (mm)
FE.Kaxial	Axial stiffness (N/mm)

#### Micro finite element analysis

To perform the µFEA analysis, the HR-pQCT images were exported and further processed using Medtool 4.5 (Dr. Pahr Ingenieurs e.U., Pfaffstätten, Austria). The image processing steps and the µFEA simulation corresponded to the methodology of Varga et al. [20]. First, the bone tissue was segmented by generating a binary 3D image, which represented bone tissue by a grey value of one and all other tissues, as well as air, by the grey value zero. Morphological filters were used to ensure that unconnected bone tissue was deleted from the image. Each voxel was then transformed into a linear hexahedral element (element side length: 82 μm). The bone material was assumed to be homogeneous, isotropic and linear elastic, with a modulus of elasticity of 15 GPa and a Poisson’s ratio of 0.3. The proximal end of the bone was fully constrained and a displacement of 0.0902 mm (1% strain relative to the specimen height) was applied at the distal end. Lateral movements at the distal end of the bone were also constrained. The µFEA models were solved with ParOSol [21] and the axial stiffness (FE.Kaxial) was calculated by dividing the reaction force by the given displacement at the distal end.

### Statistics

In accordance with the aims of this study, changes in the target parameters (Table 1) were assessed (I) between the first measurement (M1) and second measurement (M2) of the fractured radius and (II) between the ipsi- and contralateral radius at both M1 and M2. Paired, two-sided T-test were performed using custom Python scripts (SciPy [22]), to compare the parameters statistically. The general significance level was set at α = 0.05.

## Results

12 Patients (aged 40.9 ± 14 years) completed the study (Table 2). On average, the osteosynthesis material was removed after 461.2 days post-surgery. HR-pQCT measurements were performed 34.8 (3 to 119) days after plate removal (M1) and 490.5 (381 to 791) days after plate removal (M2).

**Table 2:** Characteristics of the study cohort

Patient	Age (years)	Gender	Fracture type (AO)	Dominant Side	Nicotine consumption
**1**	56	Female	C	no	no
**2**	55	Female	B	no	no
**3**	31	Female	A	yes	no
**4**	40	Male	C	yes	yes
**5**	28	Female	C	yes	no
**6**	59	Male	C	no	no
**7**	29	Male	A	yes	no
**8**	32	Male	B	yes	no
**9**	59	Female	A	yes	no
**10**	31	Female	B	no	no
**11**	50	Male	B	no	no
**12**	21	Female	B	no	yes

### Changes in the fractured side

**Fig. 3:**
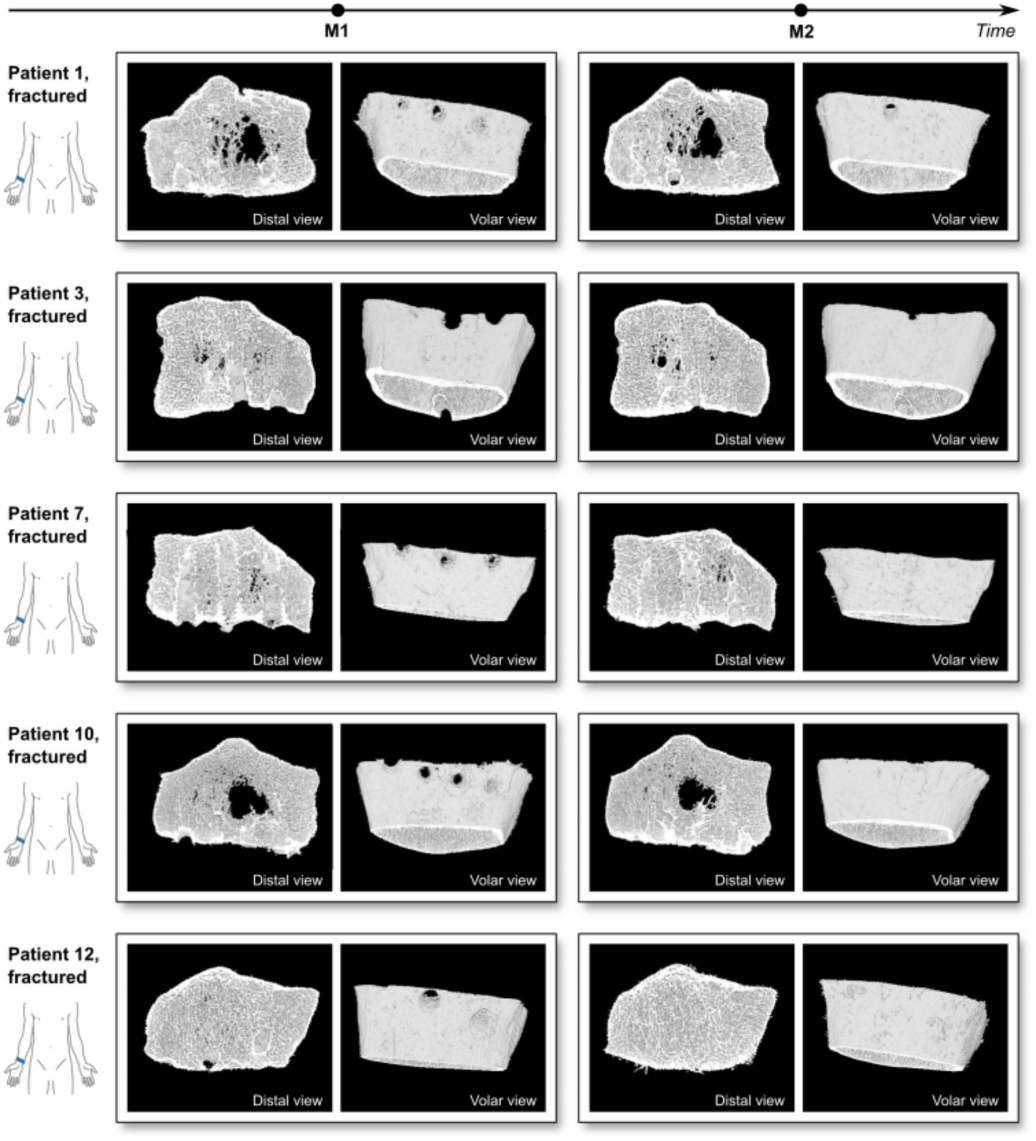
3D renderings of the HR-pQCT images of the fractured distal radius in a distal view and volar view at M1 and M2 of five patients. Large trabecular defects remained visible at M2. Residual screw holes were closed between M1 and M2 in many, but not all cases

**Fig. 4:**
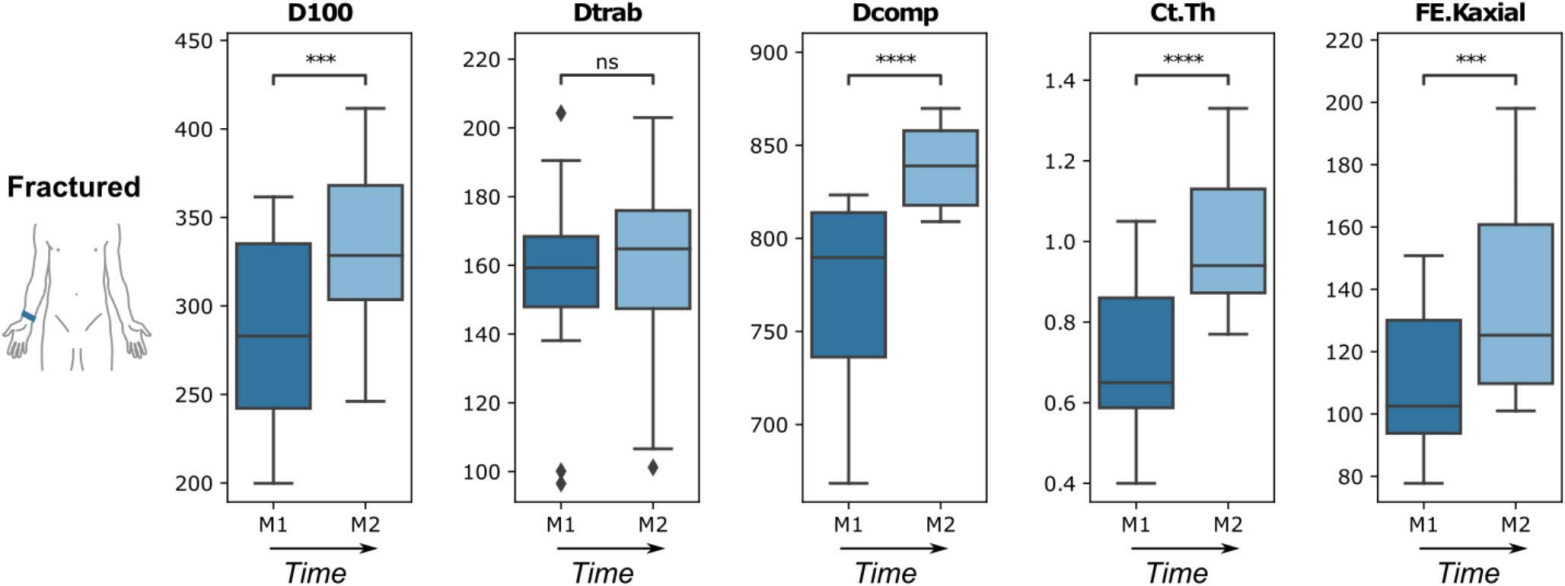
Boxplots of the target parameters of the fractured distal radius at M1 and M2. Units of the target parameters: D100, Dtrab and Dcomp in mgHA/cm^3^; Ct.Th in mm; FE.Kaxial in kN/mm. p values are indicated as not significant (ns), < 0.05 (*), < 0.01 (**), < 0.001 (***), and < 0.0001 (****)

The fracture healing process and changes in target parameters between M1 and M2 are illustrated in Figs. 3 and 4. Qualitatively, bone healing was observed at the screw holes, which were fully closed in almost all patients at M2 (Fig. 3). In some patients, trabecular defects at the centre of the radius were observed at M1, and these defects remained almost unchanged in appearance at M2. With the exception of Dtrab, there was a significant increase in all target parameters of the fractured side between the M1 and M2 (Fig. 4). Ct.Th showed the largest increase with 52%, followed by FE.Kaxial with 24%. D100 and Dcomp increased 17% and 9% respectively.

### Differences compared to healthy contralateral radius

**Fig. 5:**
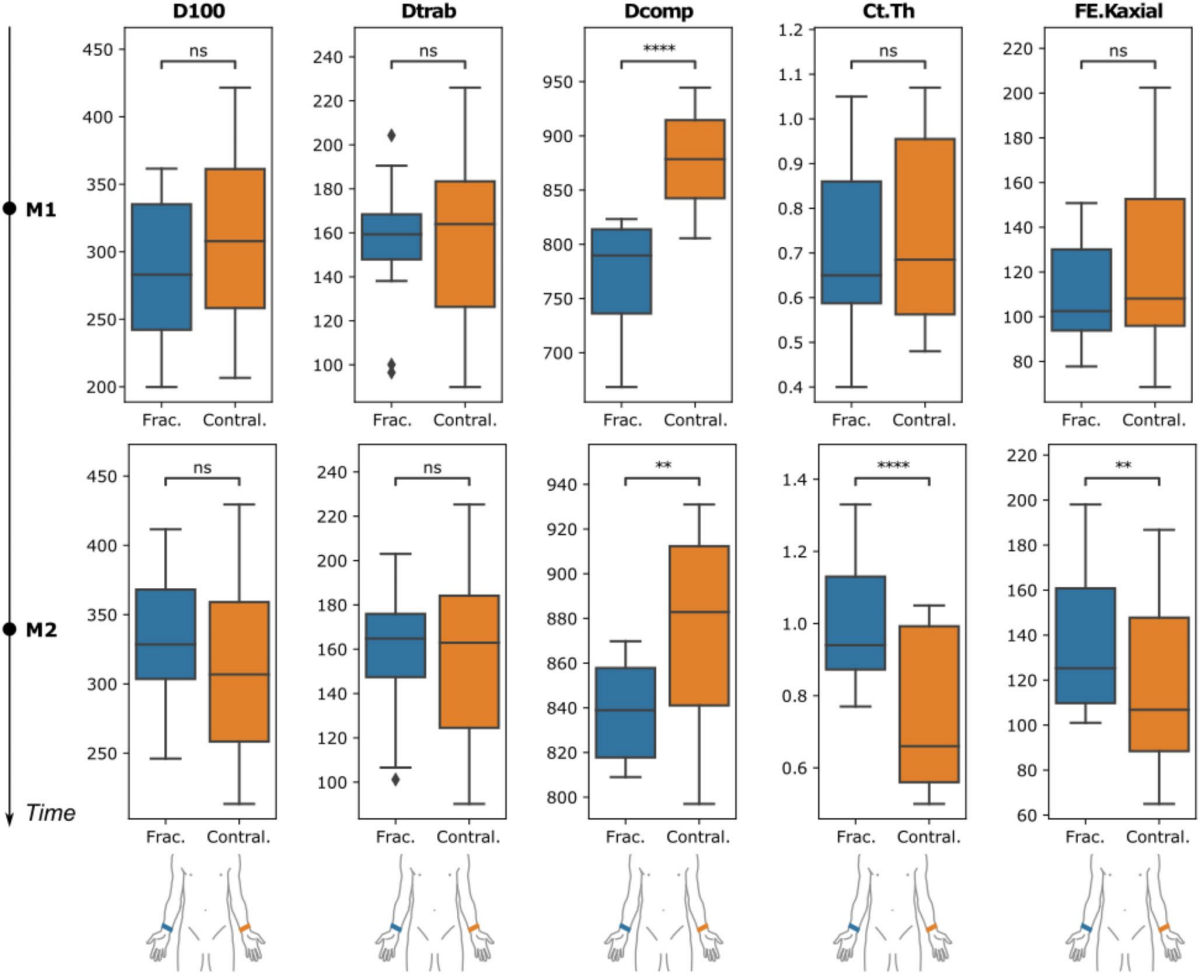
Boxplots of the target parameters at M1 (top row) and M2 (bottom row), comparing the fractured (frac.) to the contralateral (contral.) side. Units of the target parameters: D100, Dtrab and Dcomp in mgHA/cm^3^; Ct.Th in mm; FE.Kaxial in kN/mm. p values are indicated as not significant (ns), < 0.05 (*), < 0.01 (**), < 0.001 (***), and < 0.0001 (****)

At M1, a significant deficit of 12% on the fracture side could only be determined for Dcomp. At M2, the measurement showed significantly higher Ct.Th and FE.Kaxial on the fracture side by 37% and 21% respectively, while Dcomp still did not reach the level of the intact opposite side with a difference of 4%. (Fig. 5.)

## Discussion

In this study we investigated changes of microarchitecture, density and biomechanics of 12 distal radius fractures after removal of volar locking plate hardware, using HR-pQCT and µFEA. Specifically, we assessed (I) midterm changes within 16 months in the fractured radii, and (II) differences to the healthy contralateral radius at one and 16 months after plate removal.

### Changes in the fractured side

All parameters, with the exception of trabecular bone density, showed a significant change between M1 and M2. The most striking changes at the second measurement time point (M2) were an increase in cortical thickness and axial stiffness of 52% and 24% respectively.

The trabecular density showed no significant difference between M1 and M2, indicating that the trabecular modeling processes appear to have stopped. This is in agreement with qualitative observations of residual screw holes and defects in the trabecular region even at M2 (Fig. 3). While Dtrab did not change between M1 and M2, the cortical density (Dcomp) of the fractured distal radii showed an increase of 9%. These observations are consistent with findings from studies on fracture healing in humans and animals, which have shown that the cortical repair and (re)modeling processes lasted longer or took longer than was the case in the trabecular compartment [23]. 

### Differences compared to healthy contralateral radius

Concerning our second research question, interestingly, there was a significant difference in cortical density (Dcomp) at M1. Several studies report a deficit in bone mineralization following plate osteosynthesis [24–26].

A frequently discussed mechanism is the osteodegenerative processes near the plate. Due to its high material rigidity, the implant carries a considerable amount of the forces acting on it and thus mechanically shields the bone [27]. This mechanism, called stress shielding, was described in particular in connection with conventional compression plates [28]. However, more recent studies provide evidence that the use of stable-angle implants can also lead to significantly reduced bone densities in the area near the plate, due to rigid fracture fixation whereby this effect was in some cases significantly more pronounced than in patients treated with compression plates [24, 29]. However, another mechanism that has been described specifically for fracture treatment with stable-angle plate systems is the inhibition of periosteal callus formation and mineralization in the cortex areas close to the plate. The etiological assumption here was that fracture fixation was too rigid by plate osteosynthesis, which restricted interfragmentary movements and consequently the initiation of secondary fracture healing, particularly near the implant [29]. 

The fractured radii did not show significantly lower axial stiffness compared to the contralateral side at any timepoint in our study. At M2, the fractured radii even significantly exceeded the axial stiffness of the contralateral side by 21% on average. Interestingly, this is in line with Spanswick et al. [30], who showed a predominance of axial stiffness of around 10–15% compared to the contralateral side at the end of the approximately 26-week follow-up period after conservative treatment. Reaching the contralateral level of stiffness was predicted to occur approximately five months after fracture onset. Remarkably at M2, the cortical thickness of the fractured side was increased significantly by 37% compared to the healthy side. However, one possible explanation for the significant difference in cortical thickness in our study is that the screw holes were not yet fully closed and an implant-associated reduction in volumetric bone mineral density.

### Limitations

Several limitations must be stated. The first and major limitation of the present study is the small number of 12 patients. Second, the µFEA approach used to determine the axial stiffness has not been validated for previously fractured radii with screw holes. The µFEA performed in this study to determine the axial stiffness was conducted according to the methodology described by Varga et al. [20]. The lower stiffness values of Varga et al. (mean 96.5 ± 39.4 kN/mm ) compared to the healthy contralateral radii in our study (mean 120.1 ± 37.8 kN/mm ) could be due to older body donors than the patients in our study. Only axial stiffness was determined as a surrogate parameter for the restoration of load-bearing competences of the distal radius, which as a singular value does not reflect some biomechanical qualities of the bone, such as torsional and bending stiffness, as well as the fracture load. 3D printing and experimental testing could be used as an alternative to µFEA in future studies, but accurately capturing morphology and mechanical properties of trabecular bone is still a topic of ongoing research [31]. Third, the standard scan region of the first-generation HR-pQCT was not individually adapted to assess the complete fracture and screw holes. Fourth, we restricted the parameters to those considered robust considering the limited resolution of the first generation XtremeCT scanner, and the unusual morphometry of previously fractured radii and screw holes. Particularly the latter makes the evaluation of trabecular thickness and other microstructural parameters hardly comparable to other studies on healthy radii.

### Implications and outlook

This is the first study to give insights into the ongoing fracture healing process after volar plate removal. It was shown that the bone still undergoes substantial changes after plate removal, particularly by building up cortical thickness, whereas trabecular defects remain. Future studies should generally have a larger number of cases and establish more numerous and more standardized measurement times. Extending this study to a larger cohort could reveal correlations of fracture healing and outcome with several parameters such as demographics, age, and time to plate removal. In addition, using a second generation XtremeCT scanner would help to better quantify microstructure and include a larger scan region. This would also allow to apply µFEA for fracture load prediction, which could reveal the impact of residual screw holes and trabecular defects on the fracture load.

## Conclusion

After volar plate removal, cortical (re)modeling processes led to an extensive elimination of bone defects and to a thickening of the cortex. At the same time, restoration of the trabecular compartment has stopped. These observations provided the insight that the (re)modeling processes after material removal were still not complete and led to a partial exaggeration of the bone properties relative to the intact opposite side.

## Data Availability

No datasets were generated or analysed during the current study.
